# Association between the Lymphotoxin-*α* A252g Gene Polymorphism and the Risk of Sepsis and Mortality: A Meta-Analysis

**DOI:** 10.1155/2020/7936434

**Published:** 2020-08-20

**Authors:** Shujin Guo, Qiunan Zuo, Xiaohui Li, Ye He, Yutian Zhou

**Affiliations:** The Geriatric Respiratory Department, Sichuan Provincial People's Hospital, University of Electronic Science and Technology of China, Chengdu 611731, China

## Abstract

**Background:**

The association between the lymphotoxin-*α* (*LTA*) A252G polymorphism and sepsis risk has been extensively studied, but the results have been controversial. This study is aimed at investigating the overall association between the *LTA* A252G polymorphism and the risk of sepsis/septic shock and sepsis-related mortality.

**Methods:**

We searched the PubMed and EMBASE databases to identify studies that investigated the association between the *LTA* A252G polymorphism and risks of sepsis, septic shock, and mortality. The relevant data were extracted, and statistical analyses were performed using the Revman 5.0 and STATA 12 software.

**Results:**

A total of 32 publications were included in the meta-analysis. The results demonstrated that the *LTA* A252G polymorphism showed no significant association with sepsis risk (GG+GA *vs.* AA: OR = 0.92, 95%CI = 0.79–1.07, *p* = 0.27) or with sepsis shock risk (GG+GA *vs.* AA: OR = 1.01, 95%CI = 0.84–1.22, *p* = 0.91). However, in the subgroup analyzed by ethnicity, the *LTA* A252G polymorphism significantly decreased sepsis risk in the Asian population for the recessive model [GG *vs.* GA+AA: OR = 0.82, 95%CI = 0.68–0.99, *p* = 0.04] but not in the Caucasian population. Moreover, comparisons between sepsis patients who survived and those who did not suggested that the *LTA* A252G polymorphism decreases the risk of mortality [GG+GA vs. AA: OR = 0.57, 95%CI = 0.41–0.80, *p* < 0.01].

**Conclusion:**

Our results suggested that the A252G polymorphism in the *LTA* gene decreased the risk of sepsis in Asians and may reduce mortality in septic individuals.

## 1. Introduction

Sepsis is a severe condition in terms of mortality, morbidity, and the associated economic and social burden, worldwide. Despite advanced treatments, the sepsis mortality rate, which is around 20-30%, remains hard to ignore [1]. Although the pathogenesis of sepsis is complicated, several factors are known to contribute to sepsis susceptibility and these include aging, multidrug-resistant organisms, immune suppression, and invasive procedures [1]. Furthermore, an increasing number of studies suggest that host predisposition, mainly influenced by the individual's genetic variability, is closely linked with the incidence and outcome of sepsis [2]. As sepsis is potentially a damaging inflammatory response to infection, pro- and anti-inflammatory cytokines were recognized as candidate sepsis susceptibility genes. Several susceptibility genes have been identified in genome-wide association studies (GWAS) or genetic association case-control studies [3–6], and among these genes, the lymphotoxin-*α* gene (*LTA*, also termed as tumor necrosis factor-*β*) has been extensively studied.

Genotype frequency showed that the *LTA*+252 A allele frequency was the most predominant allele in most of the world populations; and the *LTA*+252 G allele was associated with the outcome of different diseases [7]. The higher level of TNFA and LTA production is associated with the mutant allele (G) [8]. LTA exerts anti-inflammatory effects and promotes normal lymphoid tissue development [9]. It has been found that *LTA* A252G polymorphism (NcoI, rs909253, the first intron) was associated with inflammatory response, including sepsis. The *LTA* A252G polymorphism has been reported as a sepsis susceptibility variant; however, the results have been inconclusive. In 2011, a meta-analysis was performed to assess the overall association between sepsis risk and the *LTA* A252G polymorphism [9]. In our study we aimed to perform an updated meta-analysis that also included subgroup analysis, as subgroup differences may affect the reliability of the conclusions. Furthermore, in the past five years, more studies have been conducted in different populations to evaluate the impact of the *LTA* A252G polymorphism on sepsis risk, and these studies should also be included. To obtain a more reliable and precise conclusion about the association between the *LTA* A252G polymorphism and sepsis/septic shock risk and sepsis-related mortality, we performed this updated meta-analysis with accurate data and current eligible studies.

## 2. Materials and Methods

### 2.1. Study Identification and Selection

We carried out a literature search in the PubMed and EMBASE databases to identify studies that investigated the association between the *LTA* A252G polymorphism and sepsis/septic shock risk and mortality, updated on July 14, 2020. The search terms used were as follows: “sepsis or severe sepsis or septic shock” in combination with “polymorphism or variant or mutation” and “lymphotoxin-*α* or LTA or tumor necrosis factor-*β* or TNF-*β*.” The inclusion criteria were as follows: (1) they were case-control genetic studies, (2) they evaluated the association between the *LTA* A252G polymorphism and sepsis/septic shock risk or mortality, and (3) the genotype distributions for cases and controls were sufficient to estimate the odd's ratio (OR) with a 95% confidence interval (95% CI). The exclusion criteria were as follows: (1) abstracts, letters, and review articles; (2) genotype frequency not shown, and (3) repeated or overlapping data.

### 2.2. Data Extraction

Two independent authors checked all the included studies and reached a consensus on every item. The following data were extracted from the included studies: author, year of publication, country of origin, ethnicity, sepsis source, sepsis definition, gene assay method, total number and distribution of genotypes, and genotyping methods.

### 2.3. Statistical Analysis

Hardy-Weinberg equilibrium (HWE) was tested using Pearson's *χ*^2^ test. A *p* value of <0.05 indicated deviation from HWE. The strength of the association between the *LTA* A252G polymorphism and sepsis risk was assessed by the odds ratio (OR) with its corresponding 95% confidence interval (95% CI). We applied a random effects (DerSimonian and Laird method) or fixed effects model (Mantel-Haenszel method) to pool the OR values according to the results of the heterogeneity examination. Heterogeneity was assessed by a *χ*^2^-based *Q* statistic and *I*^2^, and a *p* value of <0.10 was statistically significant. For *p* < 0.10, the pooled OR was calculated using a random effects model. Otherwise, a fixed effects model was used. The *I*^2^ statistic was used to estimate the degree of heterogeneity, and a value > 50% was considered an indication of a large degree of heterogeneity. The significance of the pooled OR was evaluated by a *Z*-test, and a *p* < 0.05 was statistically significant. The dominant genetic model (GG+GA vs. AA), recessive model (GG vs. GA+AA), codominant model (GG vs. AA), heterozygote model (GA vs. AA), and allele model (G *vs.* A) were used to pool ORs and assess the association of each genotype with the risk of sepsis. Subgroup analyses were performed for accordance with HWE, ethnic group, septic shock, and mortality of sepsis population.

Publication bias was assessed by Begg's funnel plots and Egger's test. Sensitivity analyses indicating the reliability of a meta-analysis were conducted to identify the potential influence of individual data sets to the pooled OR. All statistical analyses were performed using the Revman 5.0 software (Review Manager, version 5.0, the Nordic Cochrane Centre, the Cochrane Collaboration, Copenhagen, 2008) and the STATA 12.0 software (Statistical Software, Release 12.0, College Station, TX: StataCorp LP, American, 2009).

## 3. Results

### 3.1. Characteristics of Included Studies

A total of 225 studies were identified after an initial search of the PubMed and EMBASE databases. After reading the full-text, one article [10] that was included in a previous meta-analysis [9] was excluded due to unavailable data for the genotype distribution in the sepsis group. A total of and 32 articles were included in this meta-analysis [11–42] (Supplementary Figure 1). HWE was performed in the control groups, and deviation from HWE was observed in eight studies. Seven studies were performed in Asians [22, 23, 28, 29, 36, 39, 41] and 19 in Caucasians [11–16, 18–20, 24–27, 30–33, 38, 42]. Three studies were performed in children [24, 30, 40], whereas 27 studies were performed in adults [11, 13–23, 25–29, 31–33, 35–39, 41, 42]. The relationship between the *LTA* A252G polymorphism and sepsis risk was reported in 25 studies [14, 17, 19–42], while nine studies were related to septic shock risk [20–23, 29, 31, 32, 38, 40], and 21 studies investigated the association with the mortality of sepsis [11–18, 21, 24, 26, 29, 31, 32, 34, 38–41]. The characteristics, genotype, and allele distributions of each case-control study are summarized in Table 1 and Supplement Table 1.

### 3.2. Quantitative Synthesis

**Figure 1:**
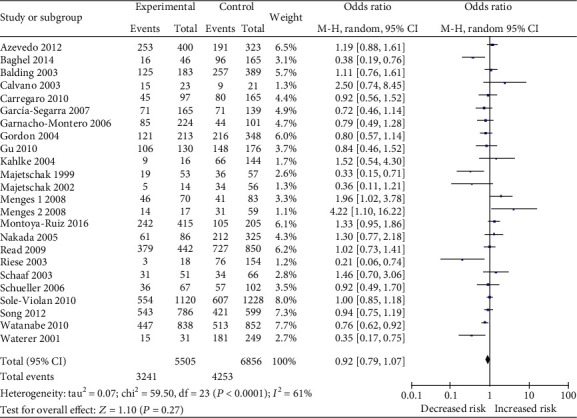
Forest plot of the association between lymphotoxin-*α* A252G (GG+GA *vs.* AA) polymorphism and sepsis risk.

For an overall analysis of sepsis risk, we analyzed the heterogeneity of GG*+*GA *vs.* AA for all 24 studies and the *χ*^2^ value was 59.5 with 23 degrees of freedom (*p* = 0.27). In addition, the *I*-square value, another index of heterogeneity, was 61%. A fixed effects model was used to pool the data. The overall OR was 0.92 (95%CI = 0.79–1.07), and the overall effect *Z* value was 1.10 (*p* = 0.27) for the GG*+*GA vs. AA model (Figure 1). The results showed that GG homozygote and GA heterozygote carriers did not increase the sepsis risk when compared with AA homozygote individuals. The results for the recessive model (GG *vs*. GA+AA), codominant model (GG *vs*. AA), and allele model (G *vs.* A), which did not indicate any associations with the risk of sepsis, are listed in Table 2.

**Figure 2:**
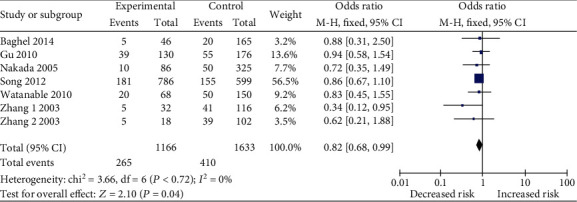
Forest plot of the association between lymphotoxin-*α* A252G (GG *vs.* GA+AA) polymorphism and sepsis risk in Asians.

For studies in accordance with HWE, no significant association was found between the *LTA* A252G polymorphism and sepsis risk (OR = 0.94, 95%CI = 0.79–1.13, *p* = 0.51 for GG*+*GA *vs.* AA). In the subgroup analysis by ethnicity (Caucasian and Asian), no association was identified between the *LTA* A252G polymorphism and sepsis risk in Caucasians (OR = 0.95, 95%CI = 0.76–1.19, *p* = 0.65 for GG*+*GA *vs.* AA) and Asians (OR = 0.84, 95%CI = 0.57–1.25, *p* = 0.39 for GG*+*GA *vs.* AA). However, the recessive model for the Asian populations showed decreased risk of sepsis (OR = 0.82, 95%CI = 0.68–0.99, *p* = 0.04 for GG *vs.* GA+AA) (Figure 2).

**Figure 3:**
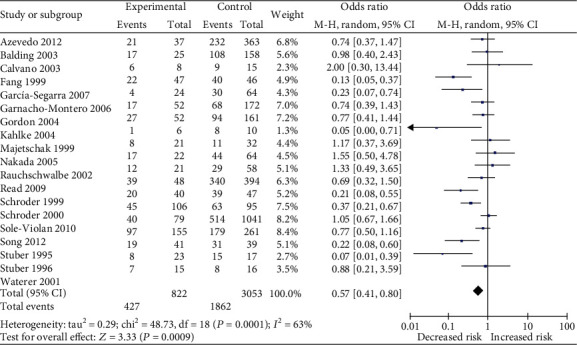
Forest plot of the association between lymphotoxin-*α* A252G (GG+GA *vs.* AA) polymorphism and sepsis mortality.

Nine studies had reported a potential effect of the *LTA* A252G polymorphism on septic shock risk [18–21, 27, 29, 30, 36, 38], while no significant association between this polymorphism and septic shock susceptibility was identified (OR = 1.01, 95%CI = 0.84–1.22, *p* = 0.91 for GG*+*GA *vs.* AA). Furthermore, a total of 21 studies had determined the association between the *LTA* A252G polymorphism and the mortality of sepsis [9–16, 19, 22, 24, 27, 29, 30, 32, 36–39], and the results of all the four models showed that the *LTA* A252G polymorphism significantly decreased the mortality risk of sepsis patients (Figure 3). A summary of all the results of statistical analysis is shown in Table 2.

### 3.3. Sensitivity Analysis and Publication Bias

**Figure 4:**
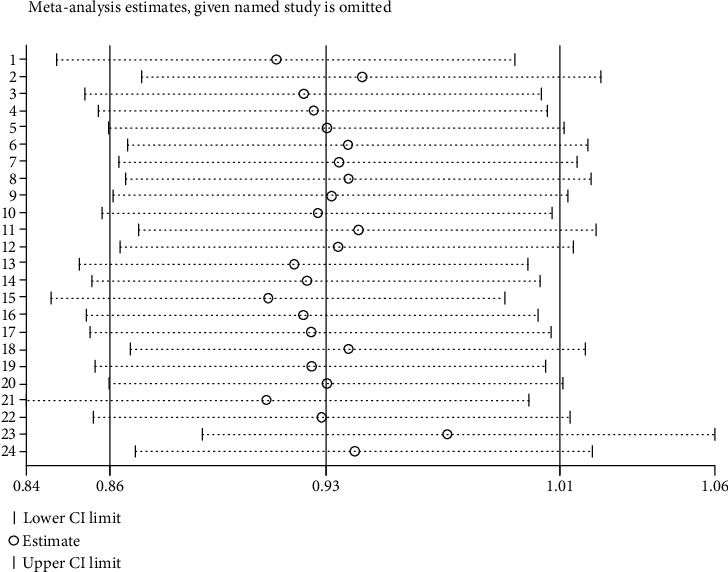
Sensitivity analysis of included studies investigated the association between lymphotoxin-*α* A252G (GG+GA *vs.* AA) polymorphism and sepsis risk.

**Figure 5:**
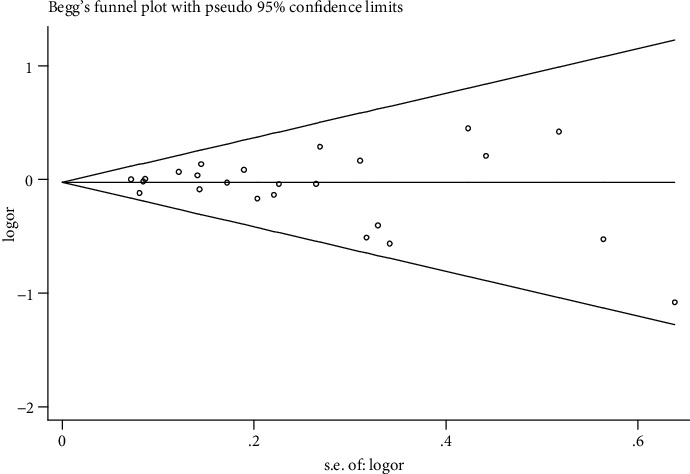
Begg's funnel plot for publication bias in selection of studies on lymphotoxin-*α* A252G (GG+GA *vs.* AA) polymorphism.

A sensitivity analysis was performed to evaluate the stability of the individual data to the pooled OR (GG*+*GA *vs.* AA). After sequentially excluding each one of the 25 studies that assessed the overall relationship between the *LTA* A252G polymorphism and sepsis risk, statistically similar results were obtained, suggesting the results of this meta-analysis were stable (Figure 4). Furthermore, similar findings were identified in other statistical models (data not shown). Moreover, publication bias was assessed by Begg's funnel plots and Egger's test. The shape of the funnel plots appeared symmetrical in the GG*+*GA *vs.* the AA comparison model, suggesting the absence of publication bias (Figure 5). Egger's test was performed to provide statistical evidence of funnel plot asymmetry. The *p* value was 0.42, indicating an absence of publication bias. In addition, no publication bias was identified in other statistical models (data not shown).

## 4. Discussion

Sepsis is a severe complication of infectious diseases which may develop to severe sepsis, septic shock or even death. Even with advanced life support and antibiotics, the mortality of sepsis is still remarkable [43]. Host genetic and immune factors play an important role in the prognosis of sepsis patients. Genetic variants can predict an individual's susceptibility to sepsis and may be helpful in determining the risk for serious complications and death in sepsis patients [43]. As inflammatory cells and cytokines are essential for the pathogenesis of sepsis, many researchers have studied polymorphisms of inflammatory cytokines. The A252G polymorphism of the *LTA* gene is one of the most studied gene polymorphisms, but the results have been conflicting. To reach a more accurate and objective conclusion, we performed this updated meta-analysis to assess the overall association between the *LTA* A252G polymorphism and sepsis risk based on current available publications. Compared with the previous meta-analysis, there are some advantages in the article. First, the article is an updated meta-analysis and included subgroup analysis. Second, the previous meta-analysis analyzed the correlation between A allele and sepsis risk. However, according to the allele frequency studies, the A allele is the predominant allele. So, we mainly analyzed the G allele in our meta-analysis. Third, we analyzed publication bias and sensitivity in our meta-analysis which was deficient in the previous article.

The meta-analysis involved 32 articles; considering the genetic background, subgroup analyses were performed for accordance with HWE, ethnic group, septic shock, and mortality of the sepsis population. The results of both overall studies and studies in accordance with HWE showed no association between the *LTA* A252G polymorphism and risk of sepsis. However, ethnicity is an important factor for the pathogenesis of sepsis, and single nucleotide polymorphisms can be used to distinguish among different ethnic populations. In terms of allele frequencies, a significant difference of G allele was found between Moroccan, African, and Asian populations; however, no difference was found in Mediterranean, European, and Japanese populations [44, 45]. In this meta-analysis, 19 of the included studies were conducted in Caucasian and seven in Asian populations. While no association was found in the Caucasian populations, in the Asian populations, the G allele was found to decrease sepsis risk, indicating the importance of ethnic differences. Only two studies included African-Americans; therefore, due to the small sample size, additional studies are needed to assess this association in the future.

Since sepsis, in severe cases, can progress to septic shock and death, we also analyzed the association between the *LTA* A252G polymorphism and the risk of septic shock and mortality. In this meta-analysis, nine studies had reported the effect of the *LTA* A252G polymorphism on septic shock susceptibility, and 21 studies had analyzed the *LTA* A252G gene variants in septic patients who survived and those who did not. The results suggested no significant effect of the *LTA* A252G polymorphism on septic shock susceptibility. The genetic distribution of GG, GA, and AA could not be extracted independently in four of the nine articles, and therefore, the negative associations for septic shock could be attributed to the small sample size. Further studies are needed for future evaluation. In the mortality analysis, all the results indicated that the *LTA* A252G polymorphism decreased the risk of sepsis-related mortality, suggesting that the presence of the G allele (GG and G) could decrease the mortality rate in septic patients.

GWAS is the most appropriate method to identify susceptible genes for sepsis [43], and many sepsis-susceptibility genes have been so far identified by GWAS [3, 4]. However, no GWAS has reported a significant association between the *LTA* A252G polymorphism and sepsis risk, indicating that this polymorphism might not have been included in those GWAS arrays. Thus, future studies are needed to further assess and validate our results.

Heterogeneity and publication bias play a determining role in the reliability of the results in a meta-analysis. Significant heterogeneity was detected in some comparisons; however, this may be due to study design differences among the included studies. When significant heterogeneity was found, a random effects model was applied for analysis. In addition, the genetic distribution of GG, GA, and AA could not be extracted independently in some cases, probably partly contributing to the existence of heterogeneity.

Publication bias and sensitivity analysis constitute an essential index for the quality and reliability of the study. Publication bias was analyzed using Begg's funnel plots and Egger's test in our study. The results indicated the reliability of our meta-analysis.

Hitherto, this is the most specific and comprehensive meta-analysis to investigate the association of the *LTA* A252G polymorphism with sepsis risk. However, this study had some limitations. First, since our literature search was conducted only in the selected databases, we might have missed relevant studies deposited in other databases. Second, since we only included published studies written in English, studies in other languages were excluded. Third, most of the included studies were conducted in Caucasian and Asian populations; therefore, the results may only be applicable to these populations. Hence, future studies are warranted to explore these associations further, particularly in African-American, African, and Latin populations. Nevertheless, this meta-analysis has made an important contribution to this field. A comprehensive evaluation of the association between the *LTA* A252G polymorphism and sepsis risk is more powerful than a single study. Furthermore, the reliability of this meta-analysis was confirmed by heterogeneity, publication bias, and sensitivity analyses.

To our knowledge, this is the most comprehensive meta-analysis to assess the relationship between the A252G polymorphism in the *LTA* gene and sepsis risk. Our results suggested that the *LTA* A252G polymorphism was significantly associated with a decreased risk of sepsis in Asian populations and with a decreased risk for mortality among septic individuals.

## Figures and Tables

**Table 1:** Characteristics of case-control studies.

Author [Ref]	Country	Ethnicity	Age	Sepsis source	Sepsis type	SNP method	HWE	Primer	BM	Sepsis definition
Stuber et al. [11]	Germany	Caucasian	>18	ICU	SS	PCR	Yes	Yes	No	Yes
Stuber et al. [12]	Germany	Caucasian	NA	ICU	SS	PCR	Yes	Yes	No	Yes
Fang et al. [13]	Germany	Caucasian	>18	ICU	SS	PCR	No	Yes	No	Yes
Majetschak et al. [14]	Germany	Caucasian	≥18	Trauma	SS	PCR	Yes	Yes	No	Yes
Schroder et al. [15]	Germany	Caucasian	>18	ICU	S	PCR	Yes	Yes	No	Yes
Schroeder et al. [16]	Germany	Caucasian	>18	SICU	SS	PCR	Yes	Yes	No	Yes
Waterer et al. [17]	American	Mixed	≥18	CAP	SS	PCR	Yes	Yes	Yes	Yes
Rauchschwalbe et al. [18]	Germany	Caucasian	>18	Surgery	S, SS	MS-PCR	Yes	Yes	No	Yes
Majetschak et al. [19]	Netherland	Caucasian	≥18	Trauma	SS	PCR-RFLP	Yes	Yes	No	Yes
Schaaf et al. [20]	Germany	Caucasian	≥18	CAP	S, SS, SSH	PCR	Yes	Yes	No	Yes
Calvano et al. [21]	American	Mixed	≥18	SICU	SH, S	PCR	Yes	Yes	Yes	Yes
Zhang 1 et al. [22]	China	Asian	≥18	ASP	SH	PCR	No	Yes	No	Yes
Zhang 2 et al. [23]	China	Asian	≥18	ASBP	SH	PCR	No	Yes	No	Yes
Balding et al. [24]	Ireland	Caucasian	Child	Sepsis	S	PCR	No	Yes	No	No
Riese et al. [25]	Germany	Caucasian	>18	Surgery	S	PCR	No	Yes	No	Yes
Kahlke et al. [26]	Germany	Caucasian	≥18	Surgery	S	PCR-RFLP	Yes	Yes	No	Yes
Gordon et al. [27]	UK	Caucasian	≥18	ICU	SS, SSH	PCR-RFLP	Yes	Yes	Yes	Yes
Nakada et al. [28]	Japan	Asian	≥18	ICU	S	PCR-RFLP	Yes	Yes	No	Yes
Watanabe et al. [37]	Japan	Asian	>18	ICU	S, SSH	PCR	NA	Yes	No	Yes
Schueller et al. [30]	Germany	Caucasian	Infant	Sepsis	S	PCR	Yes	Yes	Yes	Yes
Garnacho et al. [31]	Spain	Caucasian	>18	ICU	S, SS, SSH	PCR	Yes	Yes	No	Yes
García-Segarra et al. [32]	Spain	Caucasian	>18	ICU	S, SS, SSH	PCR	Yes	No	No	Yes
Menges et al. [33]	Germany	Caucasian	≥18	Trauma	S	PCR	Yes	Yes	Yes	Yes
Read et al. [34]	UK	Mixed	Mix	Sepsis	S	PCR	Yes	Yes	No	No
Carregaro et al. [35]	Brasil	Mixed	≥18	ICU	S, SS, SSH	Taqman	Yes	Yes	No	Yes
Gu et al. [36]	China	Asian	≥18	Trauma	S	PCR	Yes	No	No	Yes
Watanabe et al. [37]	American	Mixed	≥18	ICU	S, SS	PCR	No	No	Yes	Yes
Sole-Violan et al. [38]	Spain	Caucasian	≥18	CAP	S, SS, SSH	PCR	Yes	Yes	No	Yes
Song et al. [39]	China	Asian	≥18	Sepsis	S, SS	PCR	No	Yes	No	Yes
Azevedo et al. [40]	Brazil	Mixed	<18	ICU	S, SS, SSH	PCR-RFLP	Yes	No	No	Yes
Baghel et al. [41]	Indian	Asian	>18	Surgery	S	PCR	Yes	Yes	No	Yes
Montoya-Ruiz et al. [42]	American	Caucasian	>18	Emergency	S	PCR	Yes	Yes	No	Yes

S: sepsis; SS: severe sepsis; SSH: septic shock; NA: not available; HEW: Hardy-Weinberg equilibrium; PCR: Polymerase chain reaction; PCR-RFLP: Polymerase Chain Reaction-Restriction Fragment Length Polymorphism; BM: blind method.

**Table 2:** Summary of results from different comparative genetic models.

Comparison	Stratification	No	OR (95% CI)	*p*	*I* ^2^ (%) (*p*^∗^)	Model
GG + GA *vs.* AA	Overall	24	0.92 [0.79, 1.07]	0.27	61 (<0.01)	Random
	HWE	20	0.94 [0.79, 1.13]	0.51	60 (<0.01)	Random
	Caucasian	14	0.95 [0.76, 1.19]	0.65	62 (<0.01)	Random
	Asian	4	0.84 [1.57, 1.25]	0.39	63 (0.04)	Random
	Shock	9	1.01 [0.84, 1.22]	0.91	50 (0.05)	Fixed
	Mortality	19	0.57 [0.41, 0.80]	<0.01	63 (<0.01)	Random
GG *vs.* GA + AA	Overall	25	0.92 [0.84, 1.02]	0.12	26 (0.12)	Fixed
	HWE	18	1.01 [0.88, 1.15]	0.93	14 (0.29)	Fixed
	Caucasian	12	1.08 [0.90, 1.30]	0.39	19 (0.26)	Fixed
	Asian	7	0.82 [0.68, 0.99]	0.04	0 (0.72)	Fixed
	Shock	8	0.92 [0.70, 1.22]	0.58	38 (0.13)	Fixed
	Mortality	19	0.73 [0.57, 0.93]	0.01	28 (0.13)	Fixed
GG *vs.* AA	Overall	22	0.94 [0.79, 1.12]	0.48	39 (0.03)	Random
	HWE	18	0.99 [0.85, 1.15]	0.92	29 (0.12)	Fixed
	Caucasian	12	1.04 [0.86, 1.26]	0.70	29 (0.17)	Fixed
	Asian	4	0.84 [0.65, 1.07]	0.15	0 (0.91)	Fixed
	Shock	5	1.02 [0.71, 1.46]	0.92	39 (0.16)	Fixed
	Mortality	17	0.52 [0.31, 0.85]	0.009	56 (<0.01)	Random
G *vs.* A	Overall	22	0.94 [0.85, 1.03]	0.19	56 (<0.01)	Random
	HWE	18	0.95 [0.85, 1.07]	0.41	50 (<0.01)	Random
	Caucasian	12	0.96 [0.83, 1.12]	0.63	53 (0.01)	Random
	Asian	4	0.91 [0.80, 1.03]	0.13	27 (0.25)	Fixed
	Shock	5	0.97 [0.82, 1.13]	0.67	49 (0.10)	Fixed
	Mortality	17	0.70 [0.54, 0.90]	0.005	67 (<0.01)	Random
GA *vs.* AA	Overall	22	0.89 [0.77, 1.03]	0.13	53 (0.002)	Random
	HWE	18	0.89 [0.74, 1.06]	0.19	54 (0.003)	Random
	Caucasian	12	0.86 [0.69, 1.07]	0.18	53 (0.02)	Random
	Asian	4	0.86 [0.54, 1.36]	0.51	69 (0.02)	Random
	Shock	5	0.54 [0.18, 1,61]	0.27	94 (<0.01)	Random
	Mortality	17	0.61 [0.44, 0.86]	0.004	57 (0.002)	Random

## Data Availability

The raw data supporting the conclusions of this manuscript will be made available by the authors, without undue reservation, to any qualified researcher.

## Additional Points

*Highlights*. Hitherto, this is the most specific and comprehensive meta-analysis to investigate the association of the *LTA* A252G polymorphism with sepsis risk, septic shock, and mortality.

## Supplementary Materials

Supplementary Materials Supplementary Table 1: characteristics of case-control studies and distributions of LTA genotype and allele among sepsis patients and controls [11–42]. Supplementary Figure 1: flow chart of study inclusion.
