# Supplementary material for: Association between the Lymphotoxin-α A252g Gene Polymorphism and the Risk of Sepsis and Mortality: A Meta-Analysis
Source: Biomed Res Int. 2020 Aug 20;2020:7936434. doi: 10.1155/2020/7936434 (PMC7455838; doi:10.1155/2020/7936434)

Supplementary Table 1: Characteristics of case-control studies and distributions of LTA genotype and allele among sepsis patients and controls

							Case			Control			Case		Control	
Type	Author	Year	Country	Ethnicity	Cases	Controls	GG	GA	AA	GG	GA	AA	G	A	G	A
Sepsis	Majetschak et al[12]	1999	Germany	Caucasian	53	57	4	15	34	4	32	21	23	83	40	74
	Waterer et al[15]	2001	American	Mixed	31	249	5	10	16	54	127	68	20	42	235	263
	Majetschak et al[17]	2002	Netherland	Caucasian	14	56	4	1	9	8	26	22	9	19	42	70
	Schaaf et al[18]	2003	Germany	Caucasian	51	66	6	25	20	3	31	32	37	65	37	95
	Calvano et al[19]	2003	American	Mixed	23	21	4	11	8	2	7	12	19	27	11	31
	Zhang 1 et al[20]	2003	China	Asian	32	116	5	27		41	44	31	NA	NA	126	106
	Zhang 2 et al[21]	2003	China	Asian	18	102	5	13		39	35	28	NA	NA	113	91
	Balding et al[22]	2003	Ireland	Caucasian	183	389	32	93	58	52	205	132	157	209	309	469
	Riese et al[23]	2003	Germany	Caucasian	18	154	2	1	15	22	54	78	5	31	98	210
	Kahlke et al[24]	2004	Germany	Caucasian	16	144	4	5	7	10	56	78	13	19	76	212
	Gordon et al[25]	2004	UK	Caucasian	213	348	27	94	92	50	166	132	148	278	266	430
	Nakada et al[26]	2005	Japan	Asian	86	325	10	51	25	50	162	113	71	101	262	388
	Watanabe et al[27]	2005	Japan	Asian	68	150	20	48		50	100		NA	NA	NA	NA
	Schueller et al[28]	2006	Germany	Caucasian	67	102	10	26	31	18	39	45	46	88	75	129
	Garnacho et al[29]	2006	Spain	Caucasian	224	101	16	69	139	10	34	57	101	347	54	148
	García-Segarra et al[30]	2007	Spain	Caucasian	165	139	17	54	94	15	56	68	88	242	86	192
	Menges 1 et al[31]	2008	Germany	Caucasian	70	83	46		24	41		42	NA	NA	NA	NA
	Menges 2 et al[31]	2008	Germany	Caucasian	17	59	14		3	31		28	NA	NA	NA	NA
	Read et al[32]	2009	UK	Mixed	442	850	169	210	63	334	393	123	548	336	1061	639
	Carregaro et al[33]	2010	Brasil	Mixed	97	165	6	39	52	20	60	85	51	143	100	230
	Gu et al[34]	2010	China	Asian	130	176	39	67	24	55	93	28	145	115	203	149
	Watanabe et al[35]	2010	American	Mixed	838	852	112	335	391	151	362	339	559	1117	664	1040

	Sole-Violan et al[36]	2010	Spain	Caucasian	1120	1228	83	471	566	96	511	621	637	1603	703	1753

							Case			Control			Case		Control	
Type	Author	Year	Country	Ethnity	Cases	Controls	GG	GA	AA	GG	GA	AA	G	A	G	A
Sepsis	Song et al[37]	2012	China	Asian	786	599	181	362	243	155	266	178	724	848	576	622
	Azevedo et al[38]	2012	Brazil	Mixed	400	323	69	184	147	41	150	132	322	478	232	414
	Baghel et al[39]	2014	Indian	Asian	46	165	5	11	30	20	76	69	21	71	116	214
	Montoya-Ruiz et al[40]	2016	American	Caucasian	415	205	57	185	173	19	86	100	299	531	124	286
Septic Shock	Schaaf et al[18]	2003	Germany	Caucasian	13	66	0	7	6	3	31	32	7	19	37	95
	Calvano et al[19]	2003	American	Mixed	5	21	2	1	2	2	7	12	5	5	11	31
	Zhang 1 et al[20]	2003	China	Asian	32	116	5	27		41	44	31	NA	NA	126	106
	Zhang 2 et al[21]	2003	China	Asian	18	102	5	13		39	35	28	NA	NA	113	91
	Watanabe et al[27]	2005	Japan	Asin	41	282	14	27		93	189		NA	NA	NA	NA
	Garnacho et al[29]	2006	Spain	Caucasian	114	101	45		69	10	34	57	NA	NA	54	148
	García-Segarra et al[30]	2007	Spain	Caucasian	88	139	8	26	54	15	56	68	42	134	86	192
	Sole-Violan et al[36]	2010	Spain	Caucasian	159	1228	8	64	87	96	511	621	80	238	703	1753
	Azevedo et al[38]	2012	Brazil	Mixed	254	323	43	115	96	41	150	132	201	307	232	414
							Non-survivor			Survivor			Non-survivor		Survivor	
Mortality	Stuber et al[9]	1995	Germany	Caucasian	41	39	4	15	22	9	22	8	23	59	40	38
	Stuber et al[10]	1996	Germany	Caucasian	23	17	1	7	15	3	12	2	9	37	18	16
	Fang et al[11]	1999	Germany	Caucasian	47	46	2	20	25	10	30	6	24	70	50	42
	Majetschak et al[12]	1999	Germany	Caucasian	21	32	1	7	13	3	8	21	9	33	14	50
	Schroder et al[13]	1999	Germany	Caucasian	40	47	3	17	20	10	29	8	23	57	49	45
	Schroeder et al[14]	2000	Germany	Caucasian	106	95	8	37	61	12	51	32	53	159	75	115
	Waterer et al[15]	2001	American	Mixed	15	16	2	5	8	3	5	8	9	21	11	21

	Rauchschwalbe et al[16]	2002	Germany	Caucasian	21	58	1	11	9	3	26	29	13	29	32	84

							Non-survivor			Survivor			Non-survivor		Survivor	
Type	Author	Year	Country	Ethnity	Cases	Controls	GG	GA	AA	GG	GA	AA	G	A	G	A
Mortlity	Calvano et al[19]	2003	American	Mixed	8	15	3	3	2	1	8	6	9	7	10	20
	Balding et al[22]	2003	Ireland	Caucasian	25	158	3	14	8	29	79	50	20	30	137	179
	Kahlke et al[24]	2004	Germany	Caucasian	6	10	0	1	5	4	4	2	1	11	12	8
	Gordon et al[25]	2004	UK	Caucasian	52	161	5	22	25	22	72	67	32	72	116	206
	Nakada et al[26]	2005	Japan	Asian	22	64	5	12	5	5	39	20	22	22	49	79
	Watanabe et al[27]	2005	Japan	Asian	36	77	9	27		24	53		NA	NA	NA	NA
	Garnacho et al[29]	2006	Spain	Caucasian	52	172	17		35	68		104	NA	NA	NA	NA
	García-Segarra et al[30]	2007	Spain	Caucasian	24	64	4		20	30		34	NA	NA	NA	NA
	Read et al[32]	2009	UK	Mixed	48	394	12	27	9	157	183	54	51	45	497	291
	Sole-Violan et al[36]	2010	Spain	Caucasian	79	1041	5	35	39	78	436	527	45	113	592	1490
	Song et al[37]	2012	China	Asian	155	261	35	62	58	60	119	82	132	178	239	283
	Azevedo et al[38]	2012	Brazil	Mixed	37	363	9	12	16	60	172	131	30	44	292	434
	Baghel et al[39]	2014	Indian	Asian	8	38	0	8		5	33		NA	NA	NA	NA

NA: not available

Supplementary Figure 1. Flow Chart of study inclusion

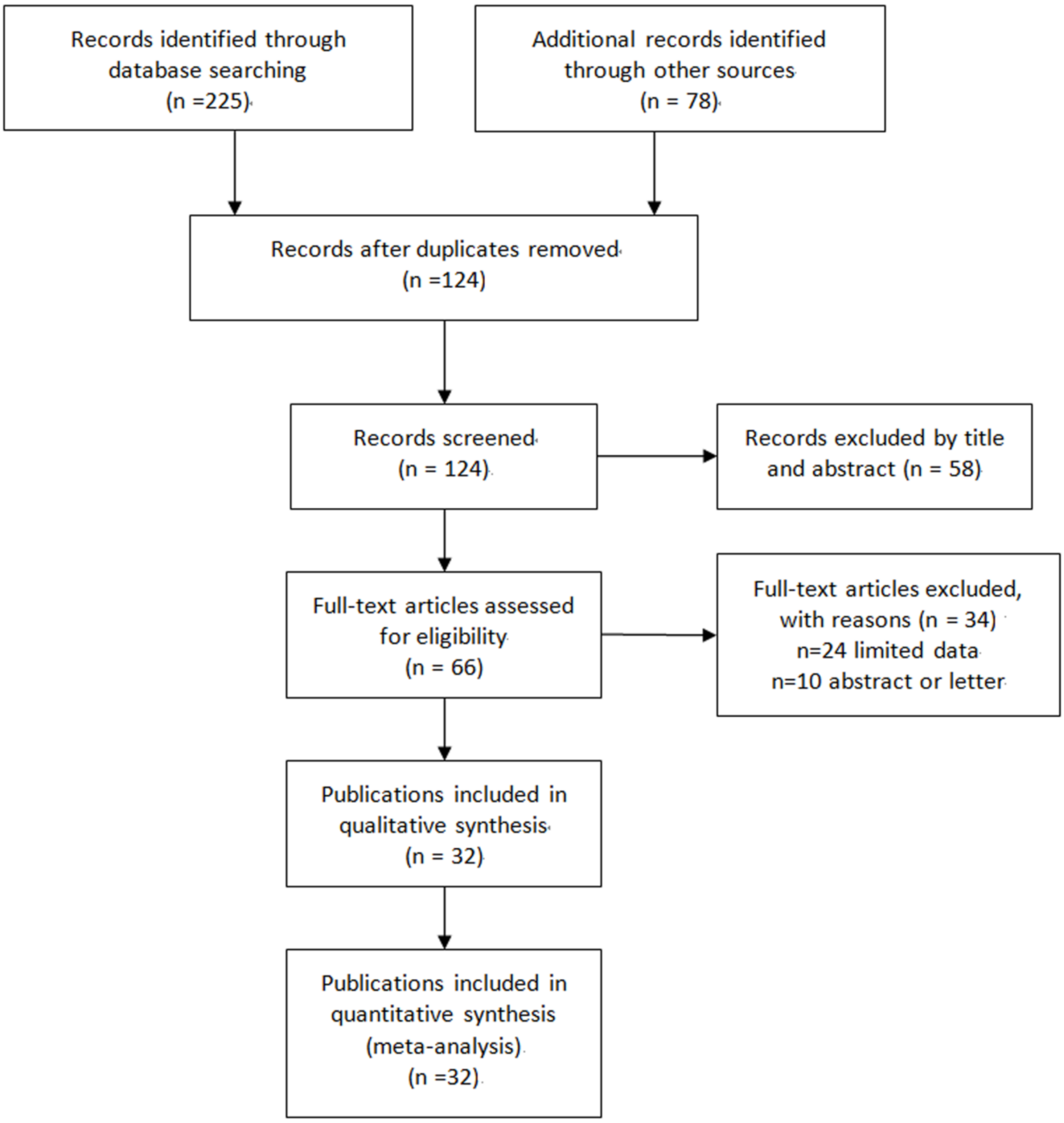

Supplement: Supplementary Materials — Supplementary Table 1: characteristics of case-control studies and distributions of LTA genotype and allele among sepsis patients and controls [11–42]. Supplementary Figure 1: flow chart of study inclusion. [file 7936434.f1.pdf]
